# Does Foraging Behaviour Affect Female Mate Preferences and Pair Formation in Captive Zebra Finches?

**DOI:** 10.1371/journal.pone.0014340

**Published:** 2010-12-15

**Authors:** Neeltje J. Boogert, Cavina Bui, Krista Howarth, Luc-Alain Giraldeau, Louis Lefebvre

**Affiliations:** 1 McGill University, Montréal, Canada; 2 Département des Sciences Biologiques, Université du Québec à Montréal, Montréal, Canada; Georgia State University, United States of America

## Abstract

**Background:**

Successful foraging is essential for survival and reproductive success. In many bird species, foraging is a learned behaviour. To cope with environmental change and survive periods in which regular foods are scarce, the ability to solve novel foraging problems by learning new foraging techniques can be crucial. Although females have been shown to prefer more efficient foragers, the effect of males' foraging techniques on female mate choice has never been studied. We tested whether females would prefer males showing the same learned foraging technique as they had been exposed to as juveniles, or whether females would prefer males that showed a complementary foraging technique.

**Methodology/Principal Findings:**

We first trained juvenile male and female zebra finches (*Taeniopygia guttata*) to obtain a significant proportion of their food by one of two foraging techniques. We then tested whether females showed a preference for males with the same or the alternative technique. We found that neither a male's foraging technique nor his foraging performance affected the time females spent in his proximity in the mate-choice apparatus. We then released flocks of these finches into an aviary to investigate whether assortative pairing would be facilitated by birds taught the same technique exploiting the same habitat. Zebra finches trained as juveniles in a specific foraging technique maintained their foraging specialisation in the aviary as adults. However, pair formation and nest location were random with regard to foraging technique.

**Conclusions/Significance:**

Our findings show that zebra finches can be successfully trained to be foraging specialists. However, the robust negative results of the conditions tested here suggest that learned foraging specializations do not affect mate choice or pair formation in our experimental context.

## Introduction

Successful foraging is essential for survival and reproductive success, as shown by Peter and Rosemary Grant's work on Darwin's finches [1]. Other research indicates that a male's foraging proficiency provides considerable direct [2]–[4] and indirect benefits [5], [6] to the choosing female. However, it is an open question whether observations of males' foraging behaviour can guide female mate choice. Instead, traits favoured in mates, such as higher courtship feeding rates [3] or brighter carotenoid-based plumage colouration [7], are assumed to be indicators of foraging ability in birds, even though there have been few, if any, studies testing for an association between these traits and foraging success. Yet there is reason to think that foraging behaviour itself might influence mate choice: the first empirical evidence of females preferring males based on foraging efficiency was provided by a recent study on red crossbills (*Loxia curvirostra*), which showed that females preferred the more efficient of two males extracting conifer seeds at different speeds [8].

Other studies suggest that females should not only pay attention to males' foraging efficiency, but also to their ability to exploit novel resources, as the latter skill may provide a means to obtain energy when regular food sources are scarce. This is best illustrated with data from the Galapagos: the only young cactus finches (*Geospiza conirostris*) to survive a severe drought were the ones that acquired the foraging skills necessary to exploit unfamiliar food sources, whereas all the juveniles that stuck with typical wet-season foraging behaviour starved to death [9]. The application of existing foraging skills to novel foods can also broaden the diet by adding profitable energy sources, as in the case of birds opening milk bottles to skim the cream underneath the lids [10], [11]. Another example is provided by New Zealand keas (*Nestor notabilis*), who started to open the lids of rubbish bins with their bills to obtain anthropogenic food scraps [12]. Comparative analyses using frequency counts of such foraging innovations in the wild (following [13]) suggest that more innovative species are more successful at establishing themselves after having been introduced by humans to novel environments [14].

The exploitation of a novel food source may benefit not only the producer, but also the individuals that manage to scrounge from these food discoveries. When animals forage in groups, joining the food discoveries of others who are specialized on exploiting different food sources will broaden the range of food types each individual can consume [15], while it permits foragers to maintain their foraging specialization and thus a higher foraging efficiency than if they would have to become generalist foragers (the ‘skill pool effect’; [16]). This principle may also apply to very small groups. For example, disassortative mating for foraging specialization might allow pair-bonded individuals to reciprocally profit from each other's foraging specializations without having to learn the other's technique, forming a skill pool based on mutual producing and scrounging. Indeed, studies that presented mated pairs with novel foraging problems in an experimental context suggest that mates can benefit from each other's food discoveries. We showed, for example, that mates of the territorial Zenaida dove (*Zenaida aurita*) of Barbados scrounged from each other's food exploitations and learned to exploit the novel food source as a result [17]. Similarly, experiments on domesticated zebra finch pairs suggest that ignorant mates can profit from their knowledgeable partners by scrounging from the latter's food exploitations of a novel foraging task [18].

Although these studies suggest that it might be profitable for animals to form pair bonds with mates that perform a foraging specialization different than their own, no one has ever tested whether female mate choice can be affected by males' foraging specializations. In this study, we test whether observation of foraging specializations can affect mate preferences in captive domesticated zebra finches (*Taeniopygia guttata*) that have been trained as juveniles to obtain their food through one of two foraging techniques. If zebra finches choose mates on the basis of foraging techniques, this would not only add to our understanding of avian mate choice, but would also be relevant to models of evolutionary divergence. The potential of behaviour to favour evolutionary change has long been recognized [19]–[24]. A recent theoretical model by van Doorn and colleagues suggests that speciation in the face of gene flow may be facilitated by females preferring males on the basis of traits that indicate adaptation to the local environment [25]. Foraging behaviour is an obvious candidate for such a trait, suggesting that female preferences for males with the appropriate foraging skills to exploit local food sources could ultimately lead to sympatric speciation.

In contrast to our first prediction that females should prefer males with different foraging specializations, the model by van Doorn et al. [25] predicts that female preferences for locally adapted males would eventually lead to positive assortative mating for the locally adaptive trait. Positive assortative mating, when combined with disruptive selection against intermediate phenotypes, plays a key role in this and other models of sympatric speciation (e.g. [26]–[28]). In birds, positive assortative mating has been established for a variety of morphological traits, such as plumage characteristics [29]–[34], bill characteristics [29], [35], [36] and body size [37], as well as age [31], [38], [39] and developmental quality [40]. With regards to behaviour, positive assortative mating has been established for explorativeness [41], [42] and vocalizations [43]–[45]. In zebra finches, previous work has reported assortative mating based on morphological and behavioural differences between zebra finch subspecies from the Australian mainland and Timor [46], between domesticated and wild populations [47], and between birds from small versus large broods [40]. In addition, mating preferences in zebra finches are substantially influenced by early learning about visual characteristics [48], including morphological novelties [49]–[51]. We predicted that, in our study, positive assortative mating for foraging techniques might occur if zebra finch mating preferences are affected by the acquisition of a novel foraging technique and exposure to its performance by conspecifics from nutritional independence until sexual maturity.

In the first of two experiments reported here, we tested whether female zebra finches taught one of two foraging techniques showed a preference for males with the same or the alternative technique in a mate choice apparatus. To remove any confounding effects of other sexually selected traits, we matched candidate mates for morphology and masked their songs. In view of the potential significance of foraging efficiency for fitness in zebra finches [52]–[55] and mate choice in red crossbills [8], we also measured the foraging performance of candidate mates and tested for its effect on female preferences.

Positive assortative mating might also be facilitated through males and females with the same foraging specialization exploiting the same food patches and thus encountering each other more often than birds with different foraging techniques. In the second experiment, we released these zebra finches in mixed-sex and mixed-foraging technique flocks into aviaries in which each side contained only foraging patches requiring one of the two foraging techniques to exploit, thus simulating different ‘microhabitats’ within the aviary. We then investigated whether pair formation and nest location were assortative or disassortative with regards to foraging technique. However, this assumes that zebra finches, when interacting with conspecifics performing the alternate foraging technique in aviary flocks, continue to perform only the technique they were trained on as juveniles, an assumption that we tested first.

## Methods

### Ethics Statement

The experiments described in this study were approved by the Animal Care Committee of the Université du Québec à Montréal, protocol #0807-592-0708 and conformed to all guidelines of the Canadian Council on Animal Care.

### UTest subjects and housing

We bred ten adult pairs from the zebra finch colony at the Université du Québec à Montréal to obtain 51 chicks to participate in our experiments. Chicks were separated from their parents at the age of 6 weeks and housed with 1–2 other birds of the same sex and similar age, but from a different family, in housing cages (57×29×42 cm) containing two perches, a tree branch and two reed nest baskets. Males and females were kept in separate rooms, illuminated with standard 40 W and wide spectrum Gro-Lux® fluorescent tubes on a 12∶12 h light∶dark cycle (lights on at 0600, off at 1800 hours) and kept at 24±2°C ambient temperature. The regular diet of vitamin-supplemented mixed millet seed, fresh water, cuttlefish bone and crushed oyster shells was supplemented once a week with fruits, vegetables and protein paste.

### Training on foraging tasks

**Figure 1:**
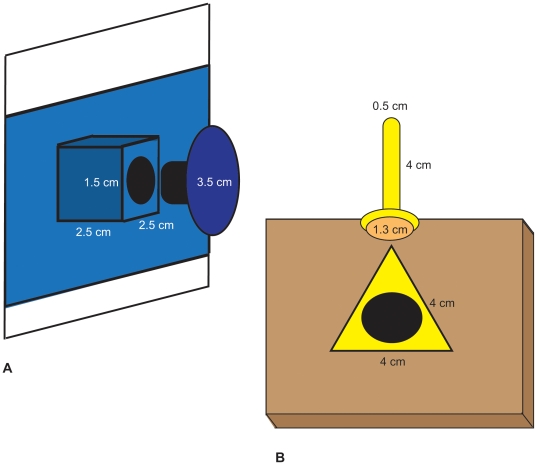
The base of the apparatus for the lid task (A) consisted of a wooden grid (26×22×2 cm) surrounded by corrugated-plastic walls (28 cm high) on three sides. Ten wooden cubes wrapped in blue tape were attached to the walls 4.5 cm from the cage floor at 4.5 cm intervals. Each cube contained one well (0.8 cm deep, 1.5 cm wide) with 2 white millet seeds, covered with a blue circular cardboard lid. The cardboard lid was lined with blue tape and pierced with a 1 cm-long screw. The screw was wrapped in foam material so that it fitted snugly into the well. To solve the task, the bird had to grab the edge of the cardboard lid with its beak and flip it out of the well. The base of the apparatus for the stalk task (B) consisted of a wooden grid (26×22×2 cm) with two rows of 5 wells (0.8 cm deep, 1.5 cm wide) spaced 4.5 cm apart. Each well opening was at the centre of a triangle of yellow tape. Each well contained 2 white millet seeds and was covered with a wooden stalk wrapped in yellow tape, to which a vinyl bumper was attached at the bottom with a pushpin. To extract the seed, the bird had to grab the stalk with its beak and pull it out of the well.

We transferred juvenile males (*N* = 18) and females (*N* = 33) when 65±5 days old (i.e. once males had acquired their songs; [56]) to visually isolated, same-sex experimental rooms where they were trained for 45 days to obtain their food by solving either a lid-flipping or a stalk-pulling task (Figure 1). Same-sex siblings were allocated to different tasks. Birds were housed with 1–2 unrelated same-sex birds trained on the same task in white corrugated-plastic cages (24.5×55×28 cm) with wire-mesh fronts and tops and four perches. Water, cuttlefish bone and crushed oyster shells were available *ad libitum* throughout the training phase. Birds were trained each day from 0800 to 1500 hours, during which time they had access to the food provided in the foraging tasks only. From 1500 hours onwards birds could feed on mixed millet seed *ad libitum* until the lights were turned off at 2000 hours.

Birds in the same training cage were separated by a wire-mesh partition each day, just before training started, to prevent them from scrounging from solving cage companions and to ensure that each bird learned the task. We used a systematic shaping procedure [57] to get the birds to learn the foraging task. All birds learned their respective task within 10 days of training. Nevertheless, all birds were exposed to foraging tasks, refilled with seeds every 60 min, as the only means to acquire food between 0800 and 1500 hours for a total of 45 days to ensure that they would memorize their task.

After this 45-day training phase, males were trained to perform their task in the mate choice apparatus, while facing a task-solving cage companion on the other side of a wire-mesh partition. Each male was trained in the mate choice apparatus until it had solved at least 8 of the 12 tasks within 10 min. Each candidate mate also served as a cage companion. To familiarize females with the apparatus, they spent the day before preference tests started in the apparatus with their cage companions.

### Female preferences for male foraging behaviour

**Figure 2:**
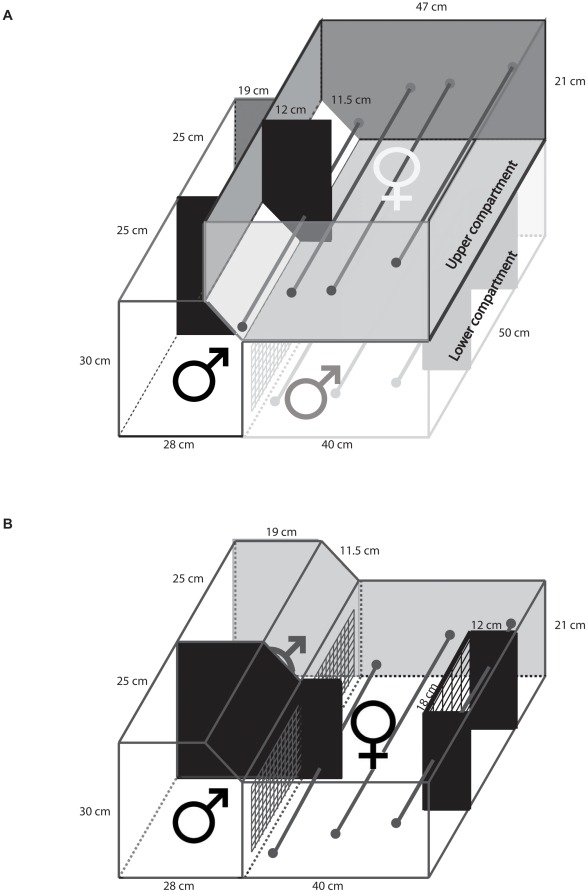
The mate choice apparatus contained two levels, an ‘upper compartment’ and a ‘lower compartment’, with two adjacent male compartments. When the female was positioned in the upper compartment for the No-Interaction test trials, she could watch two candidate mates solving their respective foraging tasks in the male compartments below her (black male symbol in (A)) through a Plexiglas window (the white 11.5×50 cm surface in the female upper compartment in (A)). The candidate mates were accompanied by their cage companions, positioned in adjacent compartments underneath the female's upper compartment (grey male symbol in (A)) and thus invisible to her. To conduct the Interaction test trials, females were positioned in the lower compartment (B) once the upper compartment and the companion males of the lower compartment (A) had been removed. For the first 10 min of the test, the female was confined to a smaller observation chamber in the back of the lower compartment. After this 10 min observation period, the female was released into the remainder of the lower compartment so that she could approach and interact with the males through the wire mesh separating her from the male compartments. Throughout the mate-choice tests, candidate mates were separated by opaque dividers.

We tested for female preferences for male foraging technique and performance, as well as for the influence of male-female interactions on female preferences for male foraging behaviour, using a 2-level mate choice apparatus. When the female was positioned in the upper compartment (Figure 2A), she could observe the males performing their respective foraging techniques but could not interact with them (‘No-Interaction tests’), whereas she could both observe and interact with the candidate mates when positioned in the lower compartment (‘Interaction tests’; Figure 2B). To test for repeatability in female preferences, each female was presented with the same two candidate mates eight times: four times while she was in the upper compartment of the mate choice apparatus (i.e. four No-Interaction test trials), and four times while she was in the lower compartment (i.e. four Interaction test trials; Table 1, Table 2).

**Table 1:** Description of the different test phases.

Test phase	Duration	Description
C: Control test	10 min10 min	Observation phase: female can see both lid and stalk tasks, but males are absentTest phase: female can see only tasks or only lids at a time
NI: No Interaction	10 min	Observation phase: female can see both males solving tasks, but males do not see female
	10 min	Test phase: female can see only one male at a time
I: Interaction	10 min	Observation phase 1: female can see both males solving tasks, and males can see female, but female cannot approach males
	10 min	Observation phase 2: female can interact with both males
	10 min	Test phase: female can interact with only one male at a time
P: Pair formation in aviary	3 days	Female is released with 3 other females and 4 males into aviary where all birds can interact freely, form pairs and build nests

**Table 2:** Timeline of the different test phases.

Day	1	2	3–6	7	8	9–12	13	14	15–18	19	20	21–27	28	29–32	33	34–37	38	39–42	43	44–47	48–50
Event	C_1_	NI_1_	T	C_2_	NI_2_	T	C_3_	NI_3_	T	C_4_	NI_4_	T	I_1_	T	I_2_	T	I_3_	T	I_4_	T	P

Capitals refer to the test phases as described in Table 1. “T” indicates continued training on the foraging tasks. Subscripts indicate the test trial numbers for the Control, No-Interaction and Interaction test phases.

Each No-Interaction test trial started with a 10-min observation phase in which the female was free to move about her upper observation compartment while the males at the lower apparatus level were solving either 12 lid tasks or 12 stalk tasks, providing access to one millet seed per task. After 10 min we scored ‘male foraging performance’ as the number of tasks each male had solved. This observation phase was followed by a 10-min test phase, for which we introduced an opaque divider in the upper female compartment that prevented the female from looking into the right male compartment when she was sitting on the perch in front of the left male compartment and vice versa (Figure 2A). During these No-Interaction tests, the candidate mates in the lower compartment were facing cage companions positioned underneath the female observation compartment. These males provided the candidate mates with company and kept the latter from being distracted by the females above. An additional measure to prevent distraction consisted of placing two natural-spectrum, 60 W light bulbs directly above each candidate mate's compartment, making it harder for the males in their brightly lit environment to detect the female in her shaded compartment above them. Thus, in the 20-min No-Interaction tests, female-male interactions were excluded. Throughout this phase, we never observed any of the candidate mates displaying to the female in the compartment above them.

To check for a female preference for one of the foraging task apparatuses, the day before each No-Interaction test trial we conducted control trials identical to test trials except that no males were placed in the lower compartment (Table 1, Table 2).

A week after a female zebra finch had gone through the series of four task control and four No-Interaction test trials she was introduced to the Interaction test. During this test she was placed in the lower compartment of the apparatus (Figure 2B) and so could interact with the candidate mates. To ensure males would continue to solve their tasks rather than courting the observing female during the first 10 min of the Interaction test, the female was confined to the rear of the observation compartment by an extra wire-mesh partition that allowed her to observe but not approach the males performing their foraging tasks. At the end of this first 10-min observation phase we recorded the number of tasks solved by each male. We then removed this extra wire-mesh partition so that the female could now approach and interact with the males through the remaining wire-mesh partition for another 10-min bout. For the final 10 min of the Interaction test trials, an opaque barrier was inserted into the female compartment that constrained the female's vision such that she could only observe the male standing on her side of the compartment and not the male in the adjacent compartment. We used females' approach behaviour towards the males' compartments during this final 10-min period for our analyses of female preferences in the Interaction tests. Each female was subjected to four consecutive Interaction test trials, using the same pair of males as in the No-Interaction test, with 5 days in between trials (Table 1, Table 2). All birds used in the mate choice apparatus were sexually naive at the time of testing.

To control for differences in the candidate males' songs, we masked their songs during all tests (including control tests) by playing a zebra finch male chorus through two Logitech R10 speakers that were attached to the sides of the two male compartments. The male chorus was composed of song recordings of the ten fathers of the birds in this study. These fathers were among the test subjects recorded in [58]. Detailed recording methods are described therein. The male chorus was created by assigning 3-min song recordings (containing natural silences between songs) of the ten fathers to three tracks in Adobe Premiere Pro CS3. We formed a single 30-min sound file by overlaying these three tracks. The result was the sound of three songs from varying combinations of males singing continuously. The same chorus was played through the two speakers at a mean of 68 dB (min: 54 dB, max: 70 dB) at 20 cm from the speaker. As the recorded males were the fathers of the test subjects, the latter had already been exposed to these songs in the breeding room during the first 65 days of their lives.

To control for morphological differences, we took a picture of each male against a white background with a Panasonic 3CCD camera and visually matched the candidate mates in each female preference test as closely as possible in terms of size, plumage morphology and beak colour. In this way we selected eight pairs of candidate males which differed in their foraging techniques but were otherwise similar. Although we initially planned to use brothers for each pair of candidate mates, we found that they differed more in morphology than did non-relatives and used non-related males to form candidate mate pairs instead. As we could form only eight stalk/lid male pairs, we had to present some pairs of candidate males to several female test subjects. We presented sisters with the same unrelated male pair to test for the influence of shared genotype and developmental conditions on female mate preferences (*N* = 31 females from 10 families, of which 15 females were trained on the stalk task and 16 females were trained on the lid task). To ensure female mate preference was not guided by hunger, the female compartment was equipped with a filled food bowl. Within each pair of candidate mates, one of the males was banded with a white leg band and the other was banded with a dark blue leg band (A.C. Hughes, Hampton Hill, U.K.). These leg band colours were chosen to be neutral in terms of attractiveness to female zebra finches [59]. The leg-band colour assigned to the lid versus stalk male in each pair was randomized. To control for side bias, we changed the sides where males performed their respective foraging techniques between each consecutive mate choice trial.

We recorded all trials from above with a Panasonic 3CCD mini-DV recorder. To measure female preferences we used the video recordings to determine the time that each female spent sitting on the perch closest to each male compartment [60] while facing that male compartment [47], a measure that has been shown to predict later pair formation [46]. Time spent on the front perch but facing backwards and time spent on other perches were excluded from analyses. All female preference tests were conducted between 0800 and 1500 hours.

### Pair formation in a heterogeneous foraging habitat

**Figure 3:**
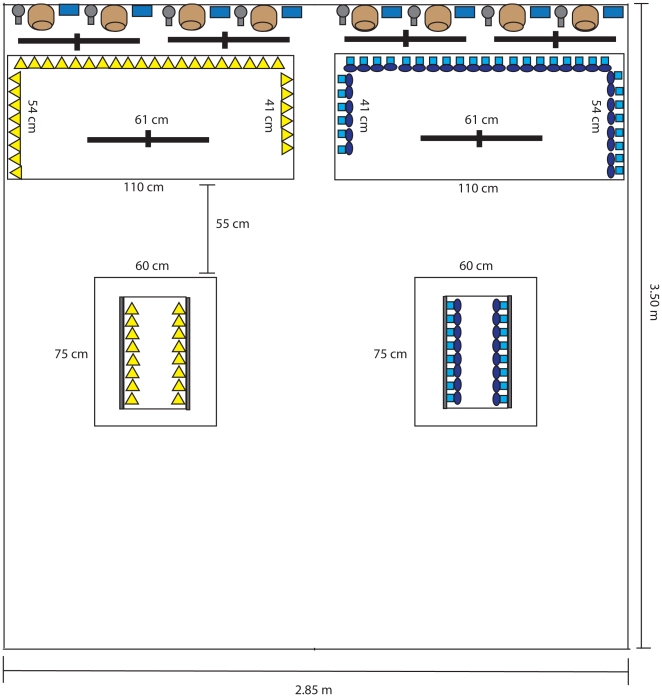
The thick black bars indicate perches, the brown cylindrical objects are nest baskets, the small blue rectangles are nest-material dispensers and the small grey circular objects are water bottles. The left side of the aviary contained the stalk habitat and the right side the lid habitat. The four white rectangles with foraging task symbols represent the foraging patches. The two patches in the middle contained 16 stalk or 16 lid tasks each, whereas the patches next to the nest boxes contained 34 stalk or 34 lid tasks each. The aviary was 2.3 m high. Foraging patches were placed on tables 90 cm above the aviary floor to facilitate the recording of all foraging occurrences and social interactions. Nest baskets, nest material dispensers and water bottles were positioned 50 cm above the tables with foraging patches.

A week after the tests of female preferences for male foraging behaviour were completed, we released the same birds in mixed-sex flocks of 4 males and 4 females in an indoor aviary that contained two different foraging patches, thus representing a heterogeneous foraging habitat. The aim of these aviary tests was to investigate whether pair formation would be assortative, due to same-skilled males and females foraging in the same habitat, or whether pair formation would be disassortative due to birds with different foraging specializations forming a skill pool. We created a lid habitat at one side of the aviary and a stalk habitat at the other. Four nest baskets, nest-material dispensers and water bottles were available on the walls next to each foraging habitat (Figure 3). We tested eight flocks of eight birds each, consisting of two stalk-pulling males, two stalk-pulling females, two lid-flipping males and two lid-flipping females. Each male was banded with two white, yellow, grey or dark blue plastic leg bands (one on each leg). The same colours were used to band females because only a limited set of colours is supposed to be neutral in terms of attractiveness [59]. However, males and females with the same leg band colours can be distinguished easily based on sexually dimorphic plumage. Wherever possible we allocated stalk/lid sisters to the same group, together with the male pairs they had been presented with in the female preference tests. As we only had eight stalk/lid male pairs during the preference tests and we used the same males for the aviary tests, some male pairs were used in multiple aviary groups, but never in two groups tested consecutively. Across the eight aviary groups, we tested 18 males (2 trained males that were not used in the mate choice apparatus because of non-matching morphology were added to the aviary groups) and 32 female zebra finches (we added two trained females to those that finished the mate choice apparatus tests (*N* = 30), to replace a female that died after the second No Interaction test trial and to form 5 groups containing 4 different females each).

Each group was released into the aviary and video-recorded for three consecutive days from 0800 to 1600 hours. Each of the 100 wells in the foraging habitats (50 stalk wells, 50 lid wells) was refilled with two millet seeds every two hours (at 0800, 1000, 1200 and 1400 hours). At 1600 hours, mixed millet seed was provided *ad libitum* until 0800 the next morning. Lights were switched off at 2000 and switched on again the next morning at 0800 hours.

For the third and final day of observations for each aviary group, we noted the foraging habitat (stalk or lid) and the identity of the solver for each occurrence of task solving. When an individual was not seen to solve at least 10 tasks during the third day, we reviewed earlier video recordings and included task solutions on previous days until the focal individual had been observed to solve a task at least 10 times. In addition, we recorded all occurrences of scrounging, along with the identity of the individual solving the task (i.e. producer) and the identity of the scrounger.

Finally, we recorded all pair formations. We determined that a pair had been formed when a male and female were repeatedly observed to be sitting together making physical contact and to be preening each other [61]. For each nest, we recorded whether it was constructed on the stalk side or the lid side of the aviary (Figure 3).

### Analyses

#### Female preferences for male foraging behaviour

To analyze female mate preferences, we calculated the proportion of time that she spent facing the stalk male's compartment in relation to the total time spent facing either compartment in the mate choice apparatus. We arcsine-square-root transformed the resulting proportions to normalize their distribution. We used this measure of female preference as a response variable in a linear mixed-effects model that contained the following fixed effects: training treatment (i.e. lid or stalk), test trial number, lid male's identity, colour of lid male's leg bands, lid male's performance during observation phase and stalk male's performance during observation phase. Random effects were female test subject's identity nested within family. Lid and stalk males were matched for morphology and always presented in the same combination to female test subjects. Thus, lid and stalk male identities were perfectly correlated and only the lid male's identity was included in the model to avoid collinearity problems. Likewise, only the colour of the lid male's leg bands was included, as it always complemented the stalk male's leg band colour: dark blue or white.

The linear mixed-effects model of female preferences was run first on data collected during the No-Interaction tests conducted in the upper compartment of the mate choice apparatus and secondly using the Interaction test data from the lower compartment. We used backward selection to obtain the minimal adequate model with a selection criterion of α = 0.05.

We used a similar linear mixed-effects model to analyze female preferences in the task control tests conducted when the female was in the upper compartment of the mate choice apparatus, facing the male compartments containing foraging tasks in the absence of the candidate mates. Fixed effects were training treatment and test number, while random effects were female identity nested within family.

We calculated the repeatability, *r* [62], of females' preferences and males' task performances across the four No-Interaction test trials and across the four Interaction test trials. Unfortunately, performance data of candidate mates presented to the first six lid females and first five stalk females subjected to the No-Interaction tests were lost, and one of the stalk females died after the second No- Interaction test trial, leading to a reduced amount of data on male performance in these tests. We checked whether the performance of lid males differed significantly from that of stalk males in the No-Interaction and Interaction tests using paired *t*-tests, and adopted the Bonferroni correction to adjust the α level of significance for multiple comparisons. In addition, we tested for significant differences between the two males' task performances during the observation phase of each female preference test trial by assigning the number of tasks each male solved to a ‘better’ or ‘worse’ category, depending on the performance of the other candidate mate in that trial. We conducted paired *t*-tests to explore whether the difference between the ‘better’ and ‘worse’ performer was significant for each of the four No-Interaction and Interaction test trials, again using the Bonferroni correction for multiple comparisons.

To test whether the time females spent facing the compartment of the better performer depended on the difference in the males' task performances, we ran another linear mixed-effects model. The response variable was the arcsine-square-root-transformed proportion of time females spent facing the compartment of the better performer, while fixed effects were test trial number, treatment, lid male's identity, colour of lid male's leg bands and the difference in the number of tasks performed by the ‘better’ and ‘worse’ performer (‘better’ minus ‘worse’). Random effects were female identity nested within family. We ran the model for both No-Interaction and Interaction test trials.

#### Pair formation in a heterogeneous foraging habitat

We used Chi-Square tests to determine whether pairing was more (dis)assortative than expected by chance, and whether technique-matched pairs were more likely to construct their nests on the side of the aviary containing their foraging task, pooling data across groups. As the formation of second and subsequent pairs in the aviary flocks is not independent of previous pair formations (the set of available mates reduces with each subsequent pair formed), we also tested whether the first pair in each aviary flock was technique-matched more often than expected by chance. We used a paired Wilcoxon signed-ranks test to assess whether, in birds that started to solve both tasks, the latency to solve the trained task differed from the latency to solve the task on which they had not been trained. Finally, we tested whether scrounging occurred more frequently in disassortative than in assortative pairs using a generalized mixed-effects model with the number of scrounging occurrences within a pair (Poisson-distributed) as the response variable and assortativeness of the pair as the predictor variable. We included male identity and flock as random effects to correct for the fact that some pairs in different aviary flocks contained the same male, and for the fact that most flocks contained several mated pairs.

We used R version 2.8.1 [63] for all our analyses.

## Results

### Female preferences for male foraging behaviour

**Figure 4:**
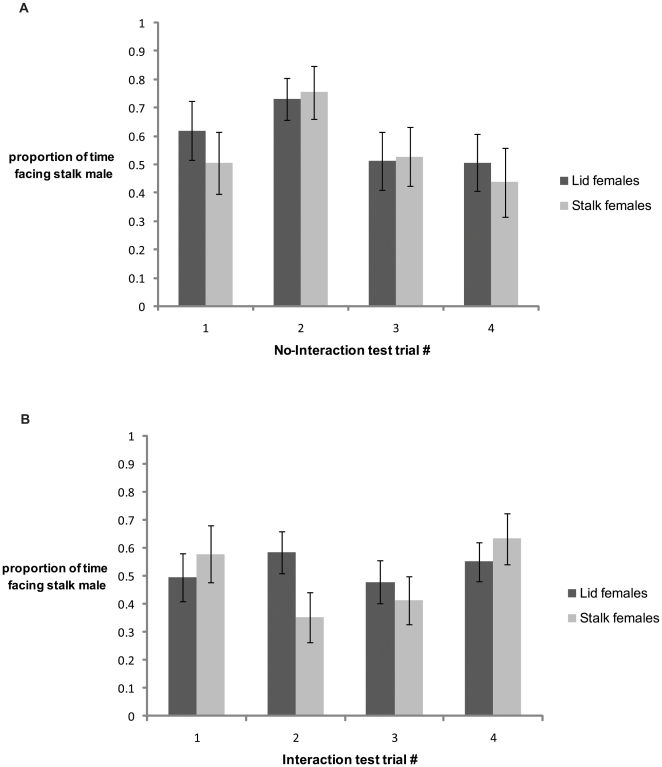
Mean proportion of time (± SE) that females trained on the lid task (*N* = 16, dark grey bars) and females trained on the stalk task (*N* = 15, light grey bars) spent facing the stalk male's compartment in each of the four (A) No-Interaction and (B) Interaction test trials. Chance proportion, indicating random choice, is 0.5.

Task control tests revealed that females, in the absence of males, were indifferent and little interested in the foraging tasks available in the mate choice apparatus. They spent no time facing either task compartment in 29 of the 122 task control tests ( = 23.8%), and neither training treatment nor test trial number predicted the proportion of time they spent in front of the stalk compartment (linear mixed-effects model, treatment: estimate ± SE = 0.033±0.071, *t*_20_ = 0.462, *P* = 0.649; test: 0.024±0.028, *t*_90_ = 0.842, *P* = 0.402). In contrast, in No-Interaction test trials all females showed interest in the task compartments. However, their prior task training had no influence on the mean proportion of time they spent facing one male or the other (Figure 4A) and the same was true for Interaction tests (Figure 4B). Females' behaviour from one trial to another was not repeatable (Table 3) and could not be predicted by any of the variables included in the model.

**Table 3:** Repeatabilities (*r*) of female preferences for stalk male's compartment.

Female training	Mean proportion of time facing stalk male (Min–Max)	Repeatability *r*	DF	*F*	*P*
**No-Interaction tests**					
Lid	0.592 (0.00–1.00)	−0.082	16, 47	0.708	0.772
Stalk	0.559 (0.00–1.00)	−0.162	14, 43	0.463	0.941
**Interaction tests**					
Lid	0.527 (0.00–1.00)	0.084	15, 47	1.363	0.205
Stalk	0.494 (0.00–1.00)	0.016	13, 42	1.065	0.413

Females did not only fail to show a preference for males that had either the same or a different foraging technique from theirs, but significant differences in males' foraging performances did not seem to affect their choice behaviour either. Despite the fact that we over-trained all males on their foraging specialization prior to the female preference tests, some males solved the maximum of 12 tasks in 10 minutes, whereas others did not solve any (Table 4). Even after adopting a Bonferroni correction for multiple comparisons, which lowered the α level of significance to 0.0125 ( =  normal α divided by 4 tests for each of the No-Interaction and Interaction test trials), there was a significant difference in the number of tasks solved in each test trial between the better and worse task performers, in the No-Interaction tests (test trial 1: *t*_19_ = 5.072, *P*<0.001; test trial 2: *t*_19_ = 6.419, *P*<0.001; test trial 3: *t*_18_ = 5.471, *P*<0.001; test trial 4: *t*_18_ = 4.558, *P*<0.001) as well as in the Interaction tests (test trial 1: *t*_28_ = 6.733, *P*<0.001; test trial 2: *t*_29_ = 6.099, *P*<0.001; test trial 3: *t*_29_ = 7.997, *P*<0.001; test trial 4: *t*_29_ = 7.378, *P*<0.001). However, the proportion of time females spent facing the better performer was not predicted by the magnitude of difference in performance between the better and worse task performer in the No-Interaction tests (linear mixed-effects model, difference in performance: 0.009±0.023, *t*_46_ = 0.397, *P* = 0.693), nor in the Interaction tests (0.004±0.015, *t*_85_ = 0.236, *P* = 0.814). Our finding that foraging performance did not affect female mate preferences contrasts with that of Snowberg and Benkman [8], who showed that 17 out of 20 red crossbill females preferred to approach the more efficient of two foragers. A power analysis using the raw data from [8] (kindly provided by L. Snowberg) suggests that, had there been an effect of foraging performance on female zebra finch preferences similar to the effect size in their study, we had the power to detect it (Snowberg and Benkman's study using the proportion of time females spent with the faster forager: effect size = 0.238, *N* = 20, SD = 0.304, α = 0.05, power of a one-sample *t* test = 0.914; our study, using the proportion of time females spent with the forager solving more tasks in Interaction test trial 1 (as males and females in [8] could interact with each other): *N* = 26, SD = 0.356; if our effect size was the same as in [8]  = 0.238, then our power to detect it  = 0.906). However, the actual effect size of our zebra finch females' preferences for the forager with the better performance was 0.036, which means we would have had to measure 1025 females for this to be significant.

**Table 4:** Repeatabilities (*r*) of male task performance.

Male Training	Mean performance: # of tasks solved (Min–Max)	Repeatability *r*	DF	*F*	*P*
**No-Interaction tests**					
Lid	7.333 (0–12)	0.354	5, 72	7.758	**<0.001**
Stalk	7.680 (1–12)	0.096	6, 71	2.130	0.060
**Interaction tests**					
Lid	7.118 (0–12)	0.318	7, 111	7.617	**<0.001**
Stalk	8.748 (0–12)	0.043	6, 112	1.735	0.119

Significant results are given in bold.

Lid males were consistent in their lid task performance (Table 4), both when they were facing their cage companions (No-Interaction tests), and when they were facing the female test subject (Interaction tests). Lid males did not differ significantly from stalk males in their task performance in the four No-Interaction test trials (Bonferroni-corrected α level of significance  = 0.0125; Mann-Whitney U tests comparing the number of tasks solved by lid males versus stalk males observed by each of 20 females; test trial 1: median # lids = 5.0, median # stalks = 7.0, *U* = 193.0, *P* = 0.848; test trial 2: median # lids = 8.5, median # stalks = 10.0, *U* = 198.5, *P* = 0.967; test trial 3: median # lids = 8.0, median # stalks = 8.0, *U* = 151.0, *P* = 0.384; test trial 4: median # lids = 9.0, median # stalks = 9.0, *U* = 178.0, *P* = 0.941). In two of the four Interaction test trials, stalk males performed significantly better than did lid males (Bonferroni-corrected α level of significance  = 0.0125; Mann-Whitney U tests comparing the number of tasks solved by lid males versus stalk males observed by each of 30 females; test trial 1: median # lids = 7.0, median # stalks = 11.0, *U* = 277.5, *P* = 0.010; test trial 2: median # lids = 8.0, median # stalks = 11.0, *U* = 324.5, *P* = 0.058; test trial 3: median # lids = 6, median # stalks = 10, *U* = 276.5, *P* = 0.009; test trial 4: median # lids = 7, median # stalks = 8, *U* = 417.0, *P* = 0.623), despite the fact that individual stalk males' performances were less repeatable across trials (Table 4).

### Pair formation in a heterogeneous foraging habitat

Assortative or disassorative pair formation based on foraging technique could occur only if flock members maintained their foraging specializations. Indeed, in five of our eight aviary groups, all birds solved only the foraging tasks on which they had been trained as juveniles. In total, 44 birds (out of the 50 birds used in the aviary groups) performed 1263 task solutions according to their taught foraging technique. In the remaining three aviary groups, six birds (four males and two females, representing 12% of our captive population) were observed to solve both tasks during the third day of observation. Three of these birds had been trained on the stalk task and the other three on the lid task. Solving of the task these birds were not trained on was very rare: it occurred in only 23 of the 2222 task solutions scored across all aviary groups, representing 1.03% of all well openings. The latency between contacting a task and solving it was significantly lower for the task these birds had been trained on (mean±SE = 1.61±0.30 s) than it was for the alternative task (4.93±1.13 s; paired Wilcoxon signed-ranks test: *Z* = −2.201, *N* = 6, *P* = 0.028). The birds were thus more efficient in solving the task of their specialisation than they were in solving the alternative task.

Across the eight aviary groups, 23 pairs were formed (mean±SE = 2.88±0.64 per group). Of these 23 pairs, 11 were mated assortatively with regards to foraging technique (five lid male – lid female pairs, six stalk male – stalk female pairs), while the remaining 12 pairs were mated disassortatively with regards to foraging technique (seven lid female – stalk male pairs, five stalk female – lid male pairs). Thus, pair formation was random with regards to foraging technique (Chi-Square test: *X*_1_^2^ = 0.044, *P* = 0.835), which was still the case when we considered only the first pair formed in each of the eight aviary flocks (assortative: three lid male – lid female pairs, one stalk male – stalk female pair; disassortative: three lid female – stalk male pairs; one stalk female – lid male pair; i.e. four assortative vs. four disassortative pairs). In these eight first pairs across the eight aviary groups, males wore grey leg bands in three of the pairs, white leg bands in two of the pairs, yellow leg bands in another two pairs and blue leg bands in the remaining pair. Formation of the first pair in each aviary group was thus random with regards to the males' leg band colours (Chi-Square test: *X*_1_^2^ = 1.0, *P* = 0.801). Of the seven nest locations we recorded for pairs in which both mates performed the same foraging technique (not all pairs had started to build nests), four nests were constructed on the side of the aviary containing tasks of the pair's foraging specialization, whereas three nests were located on the other side. There was therefore no evidence that nest location was related to assortative pairs' foraging specialisations.

**Figure 5:**
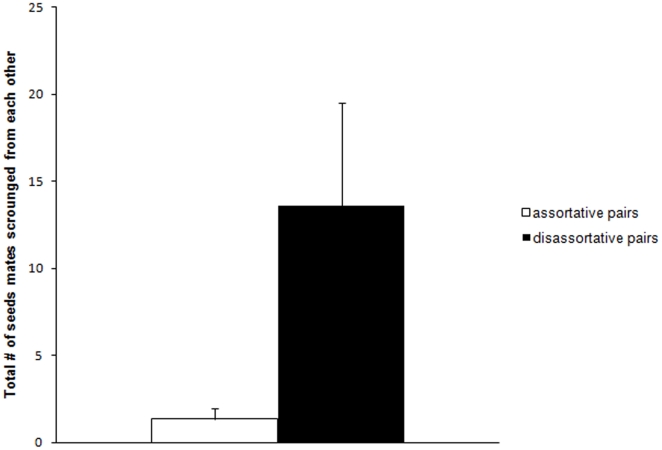
Total number of seeds mates scrounged from each other in assortative pairs (mean ± SE, *N* = 10, white bar) and disassortative pairs (*N* = 11, black bar).

Scrounging occurred significantly more often in disassortative than in assortative pairs (generalized mixed-effects model, assortativeness: estimate ± SE = −1.507±0.509, *z*_19_ = −2.962, *P* = 0.003; Figure 5). However, total food consumption did not differ between pair types (independent samples t-test: *t*_19_ = −0.192, *P* = 0.850).

## Discussion

In this study, we first tested whether female mate preferences would be affected by male foraging specializations, using a mate choice apparatus. In the second experiment, we tested whether pair formation would be assortative or disassortative with regards to mates' foraging specializations when individuals could interact freely and feed in a heterogeneous foraging habitat. We found no indication of preference based on foraging technique in the mate choice apparatus. In the mixed-sex and mixed-technique flocks in the aviary, adults retained the foraging techniques they had acquired as juveniles. Even so, pair formation was random with regards to foraging technique, and nest location was not associated with the habitat type most often exploited by the partners. It is possible that the sample size of our study did not give us enough power to detect effects that could have been present. However, our sample size is comparable to that of other zebra finch mate-choice studies reporting significant effects of morphological and developmental traits on female preferences (e.g. [40], [49], [50]) and to the crossbill study that showed mate preferences based on foraging efficiency [8]. Considering that female preferences in the mate choice apparatus and pair formation in the aviaries were completely random with regards to foraging specialization, we argue that its effect on mate choice in zebra finches is unlikely to be biologically relevant.

In our mate choice apparatus, females showed clear interest in the males while the latter foraged, but no interest in the foraging tasks *per se* when males were not present. Despite this interest in foraging males, there was no significant effect of male foraging specialization on the preferences of females when the males could court them (Interaction condition), nor when they could not (No-Interaction condition). In previous studies that reported assortative mating in zebra finches [40], [43], [47], test subjects were raised from birth by parents and/or with siblings of the type they later preferred as mates. It would be interesting to explore whether sexual imprinting on the parental foraging type would occur and result in assortative mate choice if the parents of the test subjects exploited different types of foraging patches in the nesting environment.

Consistent differences in males' foraging performance did not significantly affect female preferences either. This finding contrasts with that of Snowberg and Benkman in red crossbills [8], even though the observation and test phases of our experiment were of the same duration as in their study, and our zebra finch males differed significantly in the number of tasks solved as scored at the end of the observation period. However, unlike Snowberg and Benkman [8], we did not record each male's rate of task solving, as our main interest was in the effect of foraging technique, not foraging efficiency, on mate preferences. As males turned out to differ significantly in their foraging performance, the fact that both male foraging technique and foraging performance varied simultaneously may have provided females with conflicting information, possibly increasing the variability in their choices and adding noise to our data. However, our control trials showed that females did not show any preference for either foraging task itself and their choices were also random with regards to males' foraging techniques. It thus seems unlikely that male foraging technique should obscure an effect of foraging efficiency, if it were there. Even so, it would be worthwhile for future studies to unravel these two aspects of foraging behaviour; to look at the effect of foraging efficiency, one could present each female with two males performing the same technique but that differ in the number of tasks solved through experimental manipulation (e.g. by gluing the lids or stalks to the wells).

Even though female mate preferences did not seem to be affected by either foraging technique or foraging performance in our mate choice apparatus tests, outside our experimental context females may still choose mates that are more efficient in learning a novel foraging technique by assessing the males' songs. In a previous paper using the fathers of the birds in this study [58], we found that males with songs having a larger number of song elements were faster at learning to solve an extractive foraging task analogous to the lid-flipping task in this paper. Furthermore, previous studies have shown that female zebra finches prefer songs with a larger number of syllables [64], [65]. Although it may be difficult for females to assess various aspects of foraging behaviour through observation, they may choose good problem solvers as mates indirectly by selecting males with more complex songs. Since male song plays an important role in female mate choice in zebra finches [66], we masked song in the mate preference tests so that it could not be used as an indicator trait. A potential reason for females not to use foraging behaviour as a mate choice criterion is that it may vary depending on the availability of food in the environment, the social foraging context, hunger levels and other confounding factors. These factors could make a short sampling of foraging behaviour less reliable as an indicator of male quality than traits such as song complexity and beak colour, which reflect a male's developmental quality or condition over a more extended period of time [67], [68].

While female zebra finches did not show repeatable mate preferences using the males' foraging behaviour in the choice tests, they formed pairs within hours of release into the aviary, where they could assess male differences in song and morphology and share foraging habitats with males trained on the same foraging technique. The great majority (88%) of individuals in our zebra finch population exploited only the foraging habitat they had been trained on as juveniles; overall, only 1% of all the task solutions we analyzed involved the task not taught to the birds during their training phase. The six birds that exploited both habitat types thus exploited their non-trained habitat type only rarely and were significantly more efficient at exploiting the foraging tasks they had been trained on initially. Foraging specialisations were therefore by and large maintained during the three day aviary tests in populations where half of all individuals performed an alternative behaviour to access food, and where the two food sources were not separated by any barrier. Similarly, Beauchamp et al. [69] found that nutmeg mannikins (*Lonchura punctulata*) maintained their foraging specialisations for 10 consecutive days in flocks of four birds trained on two different foraging tasks, although pair formation was not considered in that study. However, contrary to our prediction of foraging technique-assortative pair formation through exploitation of the same foraging habitat, both pair formation and nest location were random with regards to foraging specialisation. As our main research question was whether foraging technique would affect partner choice and pair formation, we did not measure several other variables known to affect female preferences and pair formation, such as competitive interactions between males over food [70], intrasexual competition for mates [71], [72], the mate choices of other females ([73] and references therein), or male song complexity and performance [66]. A previous study showed that female zebra finches from experimentally reduced (2–3 chicks) or enlarged (5–6 chicks) broods preferred the songs of males from broods with the same brood size as their own [40]. Pair formation in our aviaries was random with regards to brood size, however (data not shown).

Considering that the majority of birds maintained their foraging specialization, it would be interesting to explore how spatial scale and variation in food availability may affect pair formation and nest location. In a significantly larger aviary where individuals would need to invest more time and effort to reach the alternative foraging habitat, foraging techniques may play a role in predicting social interactions, which could then result in more positive assortative pair formation and nest location than expected by chance. Even though zebra finches are long-distance dispersers [74], once settled in a colony individuals seem to differ in the food patches they prefer to exploit, and tend to arrive and leave food patches in small groups of the same individuals (personal observations). When we first released the zebra finches into our aviary, they tended to fly to the side that contained the familiar foraging habitat. However, they then spent the first hour interacting with their aviary companions before showing interest in the foraging habitats, despite this time in the morning being their usual foraging peak. If the habitats and associated nest sites had been spatially more segregated, birds may have spent more time in the side of the aviary containing the foraging habitat they were familiar with. Indeed, recent theoretical papers suggest that sympatric speciation might be possible through learned habitat preferences [75] and tradeoffs between adaptations to different habitats [26]. On the other hand, if in our experiment we had provided food in only one habitat type at a time, hunger may have driven more exchange between the aviary sides and one might predict that disassortative pair formation would have prevailed. Even in the current set-up, scrounging occurred more frequently in disassortative than in assortative pairs, suggesting that disassortative mate choice with regards to foraging technique can be advantageous.

In conclusion, the robust negative results of the three conditions (No-Interaction and Interaction in mate choice apparatus and aviary) tested here, added to the positive results of our earlier study [58], suggest that learned foraging does not affect zebra finch mate choice directly, but may do so indirectly via song.
